# s-ketamine enhances thalamocortical and corticocortical synaptic transmission in acute murine brain slices *via* increased AMPA-receptor-mediated pathways

**DOI:** 10.3389/fnsys.2022.1044536

**Published:** 2022-12-22

**Authors:** Matthias Bieber, Stefan Schwerin, Matthias Kreuzer, Claudia Klug, Marie Henzler, Gerhard Schneider, Rainer Haseneder, Stephan Kratzer

**Affiliations:** Department of Anesthesiology and Intensive Care Medicine, School of Medicine, Technical University Munich, Munich, Germany

**Keywords:** mechanisms of anesthesia, ketamine, thalamocortical, unconsciousness, patch-clamp, optogenetic

## Abstract

Despite ongoing research efforts and routine clinical use, the neuronal mechanisms underlying the anesthesia-induced loss of consciousness are still under debate. Unlike most anesthetics, ketamine increases thalamic and cortical activity. Ketamine is considered to act *via* a NMDA-receptor antagonism-mediated reduction of inhibition, i.e., disinhibition. Intact interactions between the thalamus and cortex constitute a prerequisite for the maintenance of consciousness and are thus a promising target for anesthetics to induce loss of consciousness. In this study, we aim to characterize the influence of s-ketamine on the thalamocortical network using acute brain-slice preparation. We performed whole-cell patch-clamp recordings from pyramidal neurons in cortical lamina IV and thalamocortical relay neurons in acute brain slices from CB57BL/6N mice. Excitatory postsynaptic potentials (EPSPs) were obtained *via* electrical stimulation of the cortex with a bipolar electrode that was positioned to lamina II/III (electrically induced EPSPs, eEPSPs) or *via* optogenetic activation of thalamocortical relay neurons (optogenetically induced EPSPs, oEPSPs). Intrinsic neuronal properties (like resting membrane potential, membrane threshold for action potential generation, input resistance, and tonic action potential frequency), as well as NMDA-receptor-dependent and independent spontaneous GABA_A_-receptor-mediated inhibitory postsynaptic currents (sIPSCs) were evaluated. Wilcoxon signed-rank test (level of significance < 0.05) served as a statistical test and Cohen’s U3_1 was used to determine the actual effect size. Within 20 min, s-ketamine (5 μM) significantly increased both intracortical eEPSPs as well as thalamocortical oEPSPs. NMDA-receptor-mediated intracortical eEPSPs were significantly reduced. Intrinsic neuronal properties of cortical pyramidal neurons from lamina IV and thalamocortical relay neurons in the ventrobasal thalamic complex were not substantially affected. Neither a significant effect on NMDA-receptor-dependent GABA_A_ sIPSCs (thought to underly a disinhibitory effect) nor a reduction of NMDA-receptor independent GABA_A_ sIPSCs was observed. Both thalamocortical and intracortical AMPA-receptor-mediated EPSPs were significantly increased.In conclusion, our findings show no evidence for a NMDA-receptor antagonism-based disinhibition, but rather suggest an enhanced thalamocortical and intracortical synaptic transmission, which appears to be driven *via* increased AMPA-receptor-mediated transmission.

## Introduction

Ketamine is a hypnotic agent used for the induction and maintenance of anesthesia. Due to its unique analgesic profile and sympathomimetic properties, ketamine has also gained major importance in emergency medicine. As adverse psychological effects like vivid dreaming or hallucinations can occur under the influence of ketamine, it is rarely administered alone. However, as these side effects are less prominent in children, ketamine is a commonly applied anesthetic agent in pediatric anesthesiology (Gropper et al., 2020). S-ketamine is an enantiomer of ketamine and is roughly two times more potent than ketamine. Despite ongoing research efforts and routine clinical use, the neuronal mechanisms underlying the anesthesia-induced loss of consciousness (AI-LOC) are still under debate.

Presumably, intact interactions between the thalamus and cortex constitute a prerequisite for the maintenance of consciousness (Alkire and Miller, 2005; Hudetz and Mashour, 2016). With exception of the olfactory pathway, somatosensory input is relayed to the cortex *via* first order thalamic nuclei. Figuratively spoken, the thalamus acts as the “gate to consciousness” and projects somatosensory input *via* excitatory thalamocortical relay neurons to the cortex. The main target for both thalamic and cortical input to the cortex is lamina IV (Feldmeyer, 2012; Grant et al., 2012). In contrast, corticothalamic projections even outnumber the thalamocortical ones. While the thalamocortical transmission is solely excitatory, the influence of the cortex on the thalamus can be excitatory as well as inhibitory *via* the reticular thalamic nucleus (Cox et al., 1997; Lam and Sherman, 2010; Ferrarelli and Tononi, 2011; Crandall et al., 2015). Moreover, the thalamus plays an essential role in corticocortical communication as it also receives cortical input and in turn projects *via* higher-order nuclei to the cortex (Sherman, 2016; Hwang et al., 2017). Anesthetics appear to impair cortical processing of information, thus governing states of consciousness (Alkire et al., 2008). For the aforementioned reasons, the thalamocortical network is generally considered one of the key neuronal targets with respect to AI-LOC.

Though anesthetics vary in their chemical composition, type of application, clinical profile, or target structures, most agents eventually converge on a common endpoint: unconsciousness (Franks, 2006). Most volatile and intravenous anesthetics cause suppressed metabolism in the thalamus, as seen in functional brain imaging studies (Bonhomme et al., 2001; Alkire and Miller, 2005). In the EEG, a slowing of frequencies and an increase in amplitudes can be observed (Lee et al., 2013; Li and Mashour, 2019). In contrast, ketamine increases metabolism in the thalamus and causes an enhancement of cortical high-frequency EEG patterns (Långsjö et al., 2004, 2005; Purdon et al., 2015). Whereas general anesthetics are thought to predominantly act *via* GABAergic pathways, it has been proposed that ketamine mainly targets NMDA-receptors (Franks and Lieb, 1994; Campagna et al., 2003; Hentschke et al., 2005; Garcia et al., 2010). More precisely, ketamine is supposed to cause disinhibition by preferentially inhibiting NMDA-receptors on inhibitory interneurons, which show a higher intrinsic baseline activity and might thus be more sensitive to inhibition (Homayoun and Moghaddam, 2007; Seamans, 2008). As another peculiarity, the dissociative state of anesthesia, commonly ascribed under the influence of anesthesia, is an anomalous state of altered consciousness markedly different from the AI-LOC of other hypnotic agents (Corssen and Domino, 1966).

The goal of this study was to characterize the effects of s-ketamine on the thalamocortical network. For this purpose, we used the whole-cell patch-clamp technique in acutely prepared murine brain slices to determine the influence of s-ketamine on evoked excitatory thalamocortical and intracortical synaptic transmission to cortical lamina IV, as well as NMDA-receptor-dependent and independent spontaneous cortical inhibition in lamina IV. We, therefore, applied optogenetic and electrical stimulation for evoked transmission.

## Materials and Methods

Experimental protocols as described were approved by the Ethical Committee on Animal Care and Use of the Government of Bavaria (Munich, Germany).

### Optogenetics

Anesthetized (midazolam 0.5 mg/kg, medetomidine 5 mg/kg, fentanyl 0.05 mg/kg) female C57Bl/6N mice with a minimum age of 4 weeks and a minimum weight of 10 grams were positioned in a stereotactic frame (Precision Stereotaxic System for small laboratory animals, TSE Systems, Bad Homburg, Germany). Mannitol 20% (0.04 ml; SERAG-WIESSNER, Naila, Gemany) was injected intraperitoneally to enhance transgene expression (Mastakov et al., 2001). Manual drill trepanation was performed 1.75 mm posterior and 1.2 mm lateral of Bregma. The injection needle (NanoFil^TM^, NF34BV 34 GA. BEVELED NEEDLE, World Precision Instruments, Sarasota, FL, USA), mounted on a microsyringe (Nanofil^TM^ Syringe, 10 μl, World Precision Instruments) and operated *via* a micromanipulator (Scientifica, P Pad Lite, East Sussex, UK), was subsequently introduced 3.5 mm from the skull surface, reaching the ventrobasal complex of the thalamus (VB). A micropump (UMP3 and Micro Syringe Pump Controller, World Precision Instruments) was employed to inject 500 nanoliters of an adeno associated virus (AAV) (diluted 2:1 with phosphate buffered saline (PBS)). We employed the AAV AAV1.hSyn.ChR2(H134R)-eYFP.WPRE.hGH (Penn Vector Core, University of Pennsylvania, Philadelphia, USA). This viral vector uses hSynapsin I as a promoter in order to exclusively cause expression in neurons. It encodes the light-sensitive cation channel Channelrhodopsin-2 (ChR2; Nagel et al., 2003) and enhanced yellow fluorescent protein (eYFP) to verify expression. The AAV is not able to replicate itself, limiting infection and expression of ChR2 in the region of stereotactical injection.

### Thalamocortical slice preparation

We allowed between 10 and 14 days for sufficient ChR2-expression. Then, the animals were decapitated under deep isoflurane anesthesia and the brains were removed atraumatically. Using a vibratome (HM 650 V, Thermo Fisher Scientific, Walldorf, Germany), 350 μm thick brain slices with preserved thalamocortical connectivity (Agmon and Connors, 1991) were prepared in ice-cold artificial cerebrospinal fluid modified for slice preparation [aCSF, containing (in mM): NaCl, 125; KCl, 2.5; NaH_2_PO_4_, 1.25; D-glucose, 25; NaHCO_3_, 25; MgCl_2_, 6; CaCl_2_, 0.5; pH: 7.4]. aCSF was continuously saturated with carbogen (95% O_2_/5% CO_2_). The slices were then incubated in an opaque storage chamber in standard aCFS [containing (in mM): NaCl, 125; KCl, 2.5; NaH2PO4, 1.25; D-glucose, 25; NaHCO3, 25; MgCl2, 1; CaCl2, 2; pH: 7.4] at 34°C for 30 min and aerated by carbogen, followed by another 30 min at room temperature (20°C–24°C).

### Electrophysiology

Whole-cell patch-clamp recordings from pyramidal neurons in lamina IV of the cortex and thalamocortical relay neurons in the thalamic ventrobasal complex were performed at room temperature (20°C–24°C), utilizing a voltage-clamp amplifier (SEC 10L; NPI Electronic, Tamm, Germany) and data acquisition software HEKA Patchmaster v2x90.1 (HEKA Elektronik, Reutlingen, Germany). Pipettes with an open tip resistance of 4–7 MΩ were used, filled with an intracellular solution containing (in mM): K-D-gluconate, 130; NaCl, 5; MgCl2, 2; HEPES, 10; EGTA, 0.5; K2-ATP, 2; Na2-GTP, 0.3; pH: 7.25. For recordings of spontaneous inhibitory postsynaptic currents (sIPSCs), we applied an intracellular solution containing (in mM): KCL, 140; NaCl, 5; Phosphocreatine-tris, 20; HEPES, 10; EGTA, 0.1; ATP-Mg^2+^, 2; Na2-GTP, 0.3; pH: 7.2. The brain slices were constantly perfused with carbogenated standard aCSF at a flow rate of 5–9 ml/min.

**Figure 1:**
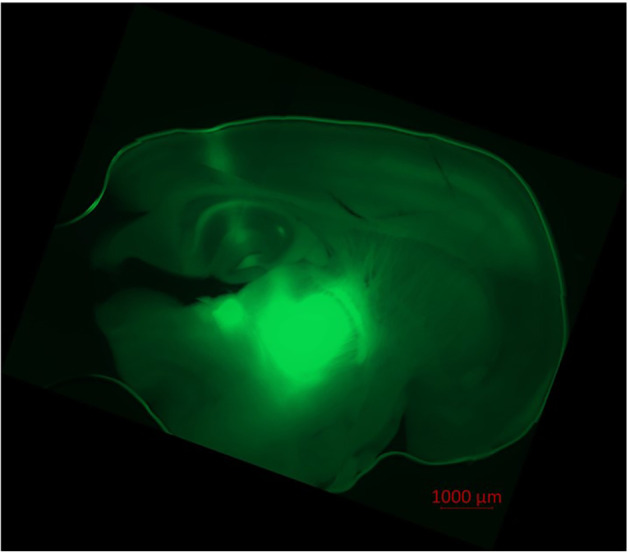
Injection of AAV1.hSyn.ChR2(H134R)-eYFP.WPRE.hGH-solution led to ChR2/eYFP expression in thalamocortical relay neurons and their projection to the cortex. Fluorescence (eYFP) is prominent in the thalamic ventrobasal complex, surrounding thalamic tissue, axons of thalamocortical relay neurons and their dentrites in cortical lamina IV, V, and VI. Slight fluorescence is observable in the hippocampus and the puncture channel. No transduced neurons were found in the cortex itself. Recorded in fluorescence microscopy (Axio Observer Z.1, fluorescence filter no. 46 HE, ZEN software, Zeiss, Oberkochen, Germany).

Pyramidal neurons in the cortex and thalamocortical relay neurons were visualized *via* infrared videomicroscopy (Axioscop FS, Zeiss, Oberkochen, Germany; Digital Camera C11440, ORCAflash4.0LT, Hamamatsu Photonics Deutschland, Herrsching am Ammersee, Germany; Dodt et al., 2002). Successful ChR2-expression was verified through successful eYFP-expression in fluorescence microscopy (fluorescence filter no. 46, Zeiss, Oberkochen, Germany; excitation wave length 488 nm), as depicted in Figure 1.

Pyramidal neurons in the cortical lamina IV were identified due to optically evoked EPSP to optogenetic stimulation of thalamocortical relay neurons and by visual control (Feldmeyer, 2012). Thalamocortical relay neurons of the thalamic ventrobasal complex were identified visually and by means of their unique electrophysiologic properties (Schwerin et al., 2020).

Excitatory postsynaptic potentials (EPSPs) were either obtained by electrical stimulation of the cortex (electrically induced EPSPs; eEPSPs) or by optogenetic activation of thalamocortical relay neurons (optogenetically induced; oEPSPs). EPSPs experiments were performed in the current clamp mode.

For eEPSPs, we used a bipolar tungsten electrode (WE2SNEX5-PT, Tungsten Concentric Electrodes, MICROPROBES, Gaithersburg, USA) that was positioned to cortical lamina II/III, using a stimulus-isolator (Stimulus Isolator, Series-No. 2533, Digitimer Ltd., Hertfordshire, UK). The electrode was triggered *via* HEKA Patchmaster, with a stimulus time of 50 μs and a stimulus energy between 0.1 and 100 V.

oEPSPs were acquired *via* optogenetic stimulation of ChR2 transduced thalamocortical relay neurons. The intensity of the continuous wave (CW) Laser (Spectra Physics Excelsior^®^ One^TM^ 488FC-70, Santa Clara, CA, USA) with a wavelength of 488 nm and a stimulus time of 25 ms was set between 1 and 70 mW *via* Excelsior^®^ One^TM^ Controller (Newport Spectra physics, Stahnsdorf, Germany, Version 3.1.1). We chose this particular pulse length in order to reduce the intensity of the laser to minimize stress and phototoxic effects on the tissue. We consider this time period as reasonable, as we conducted only single rather than repetitive stimulations.

For recordings of α-amino-3-hydroxy-5-methyl-4-isoxazolepropionic acid (AMPA)-receptor-mediated eEPSPs and oEPSPs, we added N-methyl-D-aspartate (NMDA)-receptor antagonist (2R)-amino-5-phosphonovaleric acid (AP5, 50 μM), γ-aminobutyric acid (GABA) Type B (GABA_B_)-receptor antagonist (2S)-3-[(1S)-1-(3, 4-Dichlorophenyl)ethyl]amino-2-hydroxypropyl; phenylmethyl] phosphinic acid hydrochloride (CGP 55845 hydrochloride, 5 μM) and GABA Type A (GABA_A_)-receptor antagonist bicuculline methiodide (10 μM) to the perfusate.

Recording of NMDA-receptor-mediated eEPSPs required AMPA-receptor antagonist 2, 3-Dioxo-6-nitro-1,2,3,4-tetrahydrobenzo[f]quinoxaline-7-sulfonamide disodium salt (NBQX, 5 μM), CGP 55845 hydrochloride (5 μM) and bicuculline (10 μM) in the perfusate. At resting membrane potential, the NMDA-receptor is physiologically blocked by Mg^2+^. Since this Mg^2+^-blockade is voltage-dependent (Mayer et al., 1984; Nowak et al., 1984), we applied the voltage-clamp-controlled current-clamp mode (VCcCC; Sutor et al., 2003) to set the membrane potential to −40 mV, thus dislodging the Mg^2+^-blockade while recording eEPSPs in current clamp mode. To prevent the occurrence of action potentials, sodium channel blocker lidocaine N-ethyl chloride (lidocaine, 5 mM) was added to the standard intracellular solution for these recordings.

Spontaneous GABA_A_-receptor-mediated inhibitory postsynaptic currents (NMDA-dependent GABA_A_ sIPSCs) were isolated by NBQX (5 μM) and CGP 55845 hydrochloride (5 μM) in the perfusate. Furthermore, lidocaine (5 mM) was necessitated in the intracellular solution for sIPSC-recordings to block action potential generation in the recorded cell itself. In another subset of experiments AP5 (50 μM) was added to the solution in order to exclude NMDA-receptor-dependent effects (NMDA-independent GABA_A_ sIPSCs).

Parameters of active and passive membrane biophysics, including resting membrane potential, the threshold for generation of action potentials, tonic action potential frequency, and neuronal input resistance were conducted *via* current application in steps of 10 pA to the neuron, starting at −90 pA (input resistance) over to 0 pA (resting membrane potential), continuing depolarization until the first action potential was generated (threshold for generation of action potentials) and further to +180 pA (tonic action potential frequency).

After 20 min of stable baseline recording, (S)-(+)-ketamine hydrochloride (s-ketamine, 5 μmol) was added to the perfusate. To ensure sufficient circulation and brain slice penetration of s-ketamine, recordings were repeated after 20 min.

EPSPs and parameters of active and passive membrane biophysics were analyzed using IGOR Pro 5.04B (Wave Metrics, Lake Oswego, USA). For the analysis of EPSPs we determined the area under the curve (AUC) and the amplitude of the potentials. For this purpose, we graphically averaged 10 EPSPs.

GABA_A_ sIPSCs were analyzed with Mini Analysis Program 6.0.7 (Synaptosoft, Fort Lee, USA). Parameters included frequency, AUC, and amplitude.

Salts and chemicals were purchased from Sigma–Aldrich (Steinheim, Germany), s-ketamine, CGP 55845, NBQX, and AP5 from Tocris Bioscience (Bristol, UK), midazolam from Hexal (Holzkirchen, Germany), medetomidine and fentanyl from Eurovet Animal Health (Bladel, Nederlands).

### Statistical analysis

Based on previous experience, we analyzed 6–10 slices obtained from two animals per experiment. Inclusion criteria consisted of GΩ-seal resistance, a minimum initial resting membrane potential of −60 mV in cortical cells, −50 mV in thalamic cells, and stable baseline conditions. We employed GraphPad Prism 7.03 (GraphPad Software, San Diego, USA) for statistical analysis. Wilcoxon signed-rank test was applied for simple comparisons (level of significance <0.05). We opted for a nonparametric test, as testing for normal distribution in experiments with limited sample sizes poses a challenge. Additionally, the effect size was evaluated through Cohen’s U3_1 (Cohen, 1988), hereby providing additional evidence without unduly increasing the number of animals used at the expense of animal welfare. The MATLAB-based MES toolbox (The Mathworks, Natick, MA, USA; Hentschke and Stüttgen, 2011) was used to calculate Cohen’s U3_1 with 10k-fold 95% confidence intervals. Due to the nature of these experiments, the experimenter was not blinded to the experimental conditions.

## Results

### Active and passive membrane biophysics of pyramidal neurons in cortical layer IV

As the cortical activity may be affected *via* alteration of active and passive membrane biophysics of cortical neurons, we investigated the impact of s-ketamine on the resting membrane potential, neuronal input resistance, membrane threshold for generation of action potentials, and tonic action potential frequency in pyramidal neurons in cortical lamina IV.

**Figure 2:**
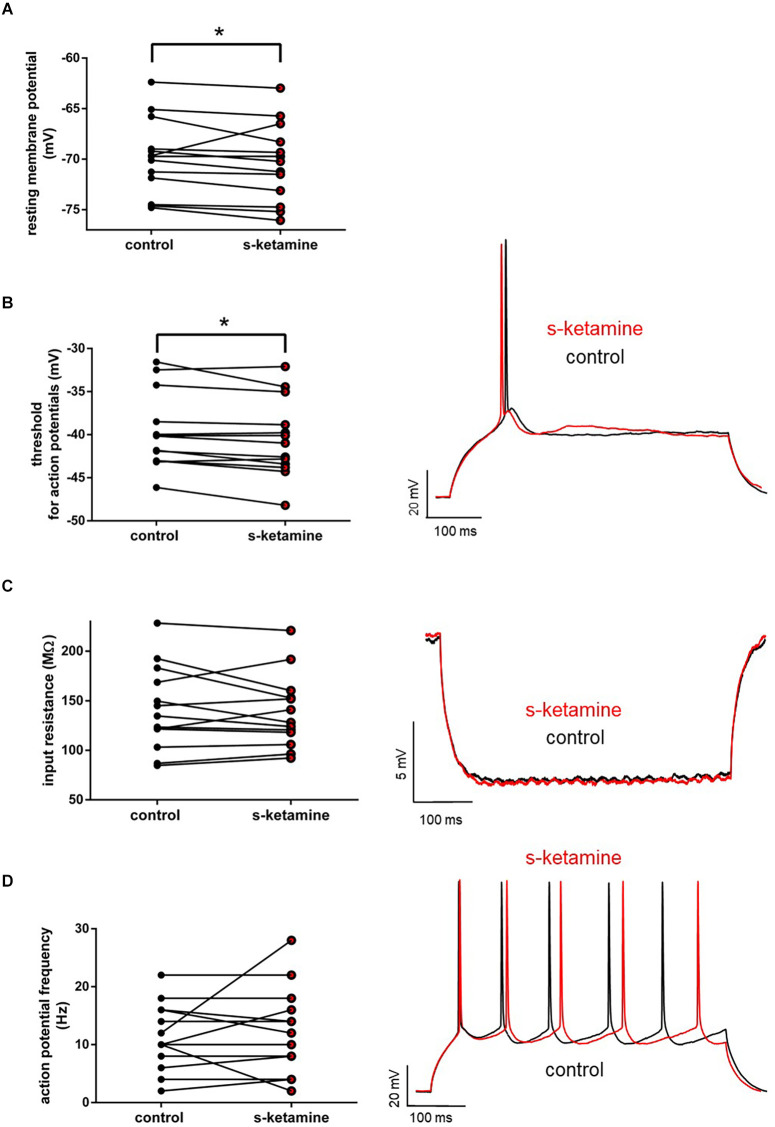
**(A)** s-ketamine marginally (<0.6 mV) but significantly (*) (*p* = 0.027) shifted the resting membrane potential towards hyperpolarization. **(B)** s-ketamine led to a signifcant (*p* = 0.013) shift of less than 0.8 mV of the membrane threshold for generation of action potential to hyperpolarization. A representative voltage trace under baseline conditions and after application of s-ketamine is shown on the right. **(C)** s-ketamine led to no significant (*p* = 0.636) alteration of the neuronal input resistence, which was conducted by applying a hyperpolarizing current (−90 pA). Representative voltage traces are shown on the right. **(D)** Application of s-ketamine did not cause significant (*p* = 0.781) change of the tonic action potential frequency, conducted *via* injecting a depolarizing current of +180 pA to the cell. Representative voltage traces are seen on the right.

S-ketamine (5 μM) did not significantly impact the input resistance in pyramidal neurons in cortical layer IV [control: 134.7 (84.7 228.4) MΩ [median (minimum maximum)] vs. s-ketamine: 128.1 (92.2 220.9) MΩ; *p* = 0.636; Cohen’s U3_1 = 0.46 (0.23 0.69) (95% CI)], nor of the tonic action potential frequency [control: 10.0 (2.0 22.0) Hz vs. s-ketamine: 12.0 (2.0 28.0) Hz; *p* = 0.781; Cohen’s U3_1 = 0.54 (0.35 0.73)]. The resting membrane potential and the membrane threshold for generation of action potentials both were significantly shifted to more negative values. Though significant (*p* = 0.013), quantification of the effect size displayed a Cohen’s U3_1 of 0.31 (0.08 0.54) to the membrane threshold with a mean difference of less than 0.8 mV [control: −40.2 (−31.6 −46.1) mV vs. s-ketamine: −41.0 (−32.0 −48.2) mV]. The mean difference of the resting membrane potential was less than 0.6 mV with a Cohen’s U3_1 of 0.15 (0 0.39) [control: −69.7 (−62.4 −74.8) mV vs. s-ketamine: −70.2 (−63.0 −76.0) mV; *p* = 0.027; *n* = 13] (Figure 2).

#### Intracortical synaptic transmission

**Figure 3:**
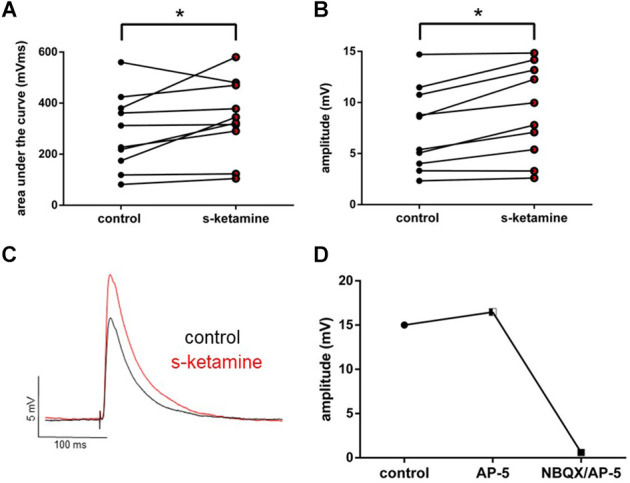
s-ketamine significantly (*) increased the AUC (*p* = 0.037) **(A)** and the amplitude (*p* = 0.004) **(B)** of intracortical eEPSPs (mixed AMPA-/NMDA-receptor mediated). Cohen’s U3_1 of AUC and the amplitude were each 0.9 (0.7 1). **(C)** Representative traces. **(D)** Adding NMDA-receptor antagonist AP-5 and AMPA-receptor antagonist NBQX led to a vanishing of intracortical oEPSPs (mixed AMPA-/NMDA-receptor mediated).

To examine the effect of s-ketamine on intracortical synaptic transmission, we conducted intracortical eEPSPs, which were electrically evoked in lamina II/III and recorded in lamina IV of the cortex. Here s-ketamine caused significant enhancement after 20 min of both analyzed parameters AUC (*p* = 0.037) and amplitude (*p* = 0.004) of intracortical eEPSPs. Cohen’s U3_1 validated a large effect size of the AUC [Cohen’s U3_1 = 0.9 (0.7 1)] and the amplitude [Cohen’s U3_1 = 0.9 (0.7 1)]. The AUC rose from 270 (82 560) mVms to 334 (105 580) mVms and the amplitude from 7.0 (2.3 14.7) mV to 8.9 (2.6 14.8) mV (*n* = 10). We performed proof-of-concept recordings by adding the NMDA-receptor antagonist AP-5 and the AMPA-receptor antagonist NBQX to the perfusate (Figure 3).

#### Thalamocortical synaptic transmission

**Figure 4:**
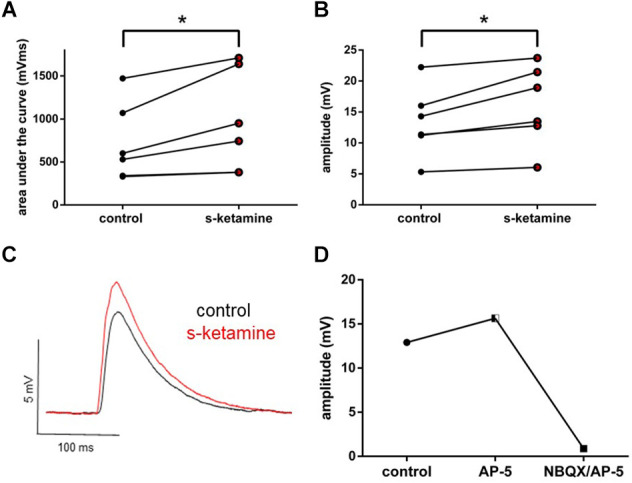
s-ketamine led to a significant (*) increase of the AUC (*p* = 0.031) **(A)** and the amplitude (*p* = 0.031) **(B)** of thalamocortical oEPSPs (mixed AMPA-/NMDA-receptor mediated). Cohen’s U3_1 was 1 (1 1) for the AUC and the amplitude. **(C)** Representative traces. **(D)** Adding NMDA-receptor antagonist AP-5 and AMPA-receptor antagonist NBQX led to a vanishing of thalamocortical oEPSPs (mixed AMPA-/NMDA-receptor mediated).

Thalamocortical oEPSPs were optogenetically evoked in ChR2 transduced thalamocortical relay neurons and recorded in downstream pyramidal neurons in the cortical lamina IV. Analogously, s-ketamine increased the AUC (*p* = 0.031) from 565 (330 1,472) mVms to 847 (380 1,711) mVms and the amplitude (*p* = 0.031) from 12.8 (5.3 22.2) mV to 16.2 (6.1 23.7) mV (*n* = 6) of thalamocortical oEPSPs significantly. Both parameters showed, according to Cohen’s U3_1, a large effect size of 1 (1 1) each. To prove that we did not directly stimulate cortical neurons by ChR2-mediated excitation we performed a recording with the NMDA-receptor antagonist AP-5 and the AMPA-receptor antagonist NBQX. In this proof-of-concept recording, no relevant remaining thalamocortical oEPSP could be observed (Figure 4).

#### Active and passive membrane biophysics of thalamocortical relay neurons in the thalamic ventrobasal complex

**Figure 5:**
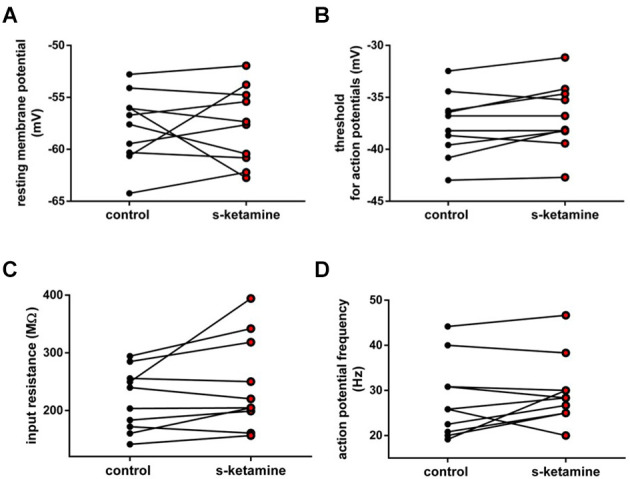
**(A)** In the presence of s-ketamine no significant alteration of the resting membrane potential of thalamocortical relay neurons could be determined (*p* = 0.770). **(B)** No significant difference of the threshold for generation of action potentials under s-ketamine was detected (*p* = 0.074). **(C)** s-ketamine did not significantly change the neuronal input resistence, which was conducted by applying a hyperpolarizing current (−90 Pa; *p* = 0.106). **(D)** Application of s-ketamine did not cause significant change of the tonic action potential frequency, conducted *via* injecting a depolarizing current of +180 pA to the cell (*p* = 0.273).

Additionally, we investigated the intrinsic properties of upstream thalamocortical relay neurons in the thalamic ventrobasal complex. S-ketamine did not significantly change the resting membrane potential [control: −57.2 (−52.8 −64.2) mV vs. s-ketamine: −57.5 (−51.9 −62.8) mV; *p* = 0.770; Cohen’s U3_1 = 0.5 (0.2 0.8)], the input resistance [control: 221.7 (141.3 294.3) MΩ vs. s-ketamine: 212.3 (156.6 394.4) MΩ; *p* = 0.106; Cohen’s U3_1 = 0.7 (0.4 1)], the membrane threshold for generation of action potentials [control: −37.5 (−32.5 −43.0) mV vs. s-ketamine: −37.5 (−31.2 −42.7) mV; *p* = 0.074, Cohen’s U3_1 = 0.75 (0.5 1)], or the tonic action potential frequency [control: 25.8 (19.2 44.2) Hz vs. s-ketamine: 28.3 (20.0 46.7) Hz; *p* = 0.273; Cohen’s U3_1 = 0.6 (0.3 0.9); *n* = 10] of thalamocortical relay neurons in the ventrobasal complex of the thalamus (Figure 5).

#### NMDA-receptor-mediated intracortical synaptic transmission

**Figure 6:**
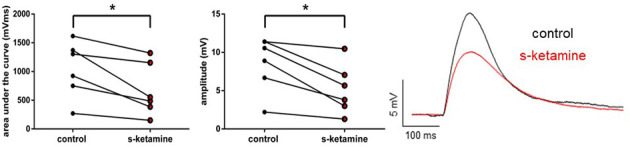
s-ketamine significantly (*) decreased the AUC (*p* = 0.031) and the amplitude (*p* = 0.031) of intracortical NMDA-receptor mediated eEPSPs. Cohens’s U3_1 was each 0 (O O). Representative traces are shown on the right.

As s-ketamine is considered to act on NMDA-receptors, we explored its effect on the NMDA-receptor-mediated intracortical synaptic transmission. NMDA-receptor-mediated intracortical eEPSPs were decreased significantly and with a quantified large effect size. In the presence of s-ketamine the AUC of NMDA-receptor-mediated intracortical eEPSPs was reduced from 1,115 (270 1,617) mVms to 521 (150 1,322) mVms (*p* = 0.031), the amplitude from 9.7 (2.2 11.4) mV to 4.7 (1.3 10.5) mV (*p* = 0.031; *n* = 6). Cohen’s U3_1 yielded a large effect size of 0 (0 0) for both parameters (Figure 6).

#### Spontaneous GABA_A_-receptor-mediated inhibitory postsynaptic currents

**Figure 7:**
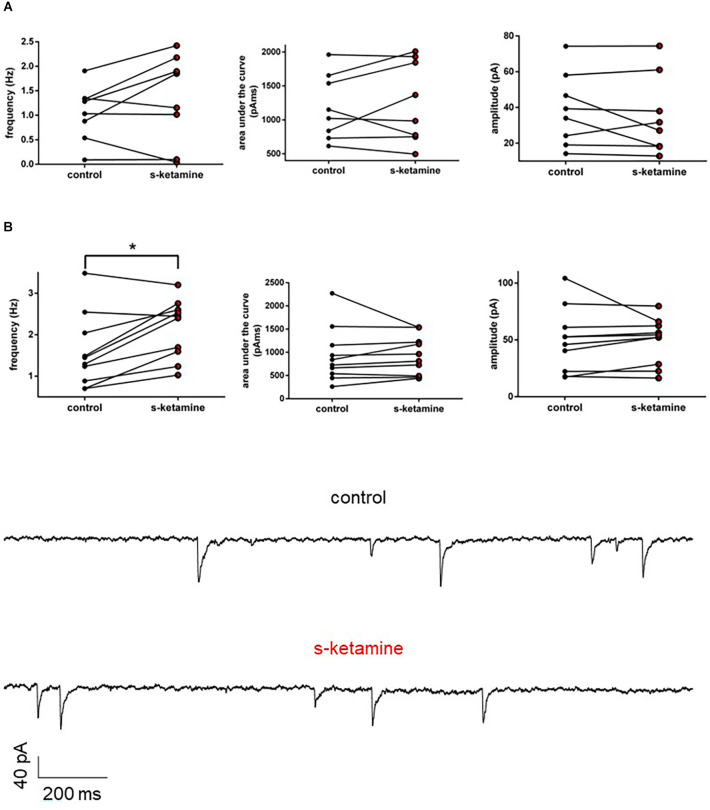
**(A)** s-ketamine had no significant influence to the frequency (*p* = 0.250), AUC (*p* = 0.844) or amplitude (*p* = 0.461) of NMDA-receptor dependent GABA_A_ slPSCs (recordings without AP-5). **(B)** s-ketamine led to a marginal (0.5 Hz), but significant (*) difference of the frequency (*p* = 0.001) of NMDA-receptor independent GABA_A_ slPSCs (recordings with AP-5). The effect size of Cohen’s U3_1 was 0.8 (0.5 1). Besides of that, s-ketamine had no significant influence to the AUC (*p* = 0.275) or the amplitude (*p* = 0.375) of NMDA-receptor independent GABA_A_ slPSCs. Representative traces are depicted below.

Frequency, AUC, and amplitude of NMDA-receptor-dependent GABA_A_ sIPSCs in cortical lamina IV remained essentially unaltered [frequency: control: 1.2 (0.1 1.9) Hz vs. s-ketamine: 1.5 (0.0 2.4) Hz; *p* = 0.250; Cohen’s U3_1 = 0.63 (0.25 0.88); AUC: control: 1,087 (615 1,962) pAms vs. s-ketamine: 1,176 (496 2,009) pAms; *p* = 0.844; Cohen’s U3_1 = 0.5 (0.13 0.88); amplitude: control: 36.7 (14.2 74.3) pA vs. s-ketamine: 29.4 (12.8 74.4) pA; *p* = 0.461; Cohen’s U3_1 = 0.38 (0.13 0.75); *n* = 8]. To exclude that NMDA-receptor-dependent effects might have disguised a potential effect of s-ketamine on GABA_A_-sIPSCs, we repeated the measurements in the presence of the selective NMDA-receptor antagonist AP5 (50 μM). Under these conditions, s-ketamine did not cause significant alteration of the AUC or the amplitude of NMDA-receptor independent GABA_A_ sIPSCs in pyramidal neurons in cortical layer IV [AUC: control: 783 (262 2,272) pAms vs. s-ketamine: 888 (438 1,539) pAms; *p* = 0.275; Cohen’s U3_1 = 0.7 (0.4 1); amplitude: control: 49.3 (17.3 104.1) pA vs. s-ketamine: 53.3 (16.4 79.7); *p* = 0.375; Cohen’s U3_1 = 0.7 (0.4 1); *n* = 10]. The frequency showed a marginal, albeit significant (*p* = 0.010) mean difference of 0.5 Hz, Cohen’s U3_1 was 0.8 (0.5 1) [control: 1.370 (0.7020 3.484) Hz vs. s-ketamine: 2.442 (1.027 3.201) Hz; *n* = 10] (Figure 7).

#### AMPA-receptor-mediated intracortical synaptic transmission

**Figure 8:**
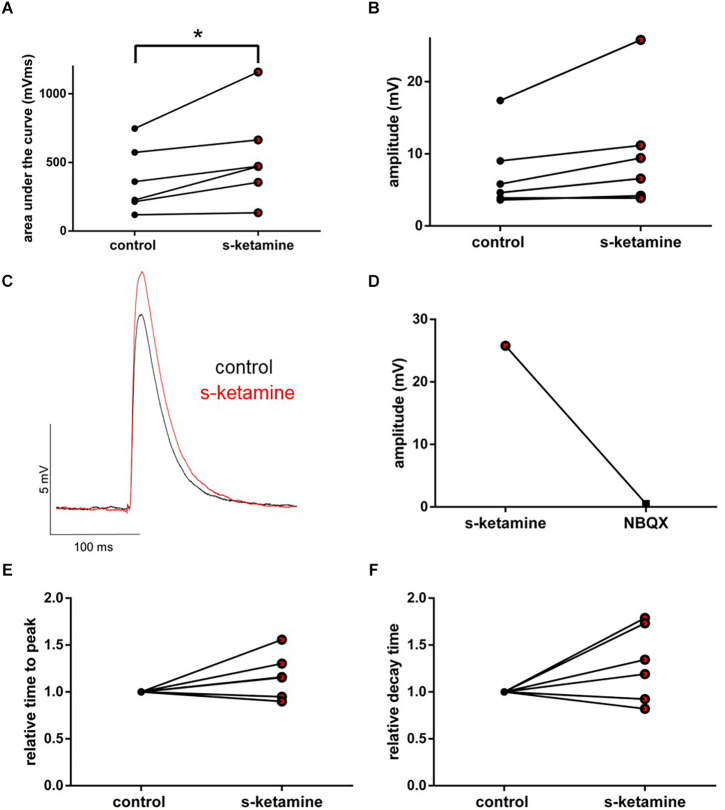
s-ketamine caused an increase of the AUC (*p = 0.031) **(A)** and the amplitude (*p* = 0.063) **(B)** of intracortical AMPA-receptor mediated eEPSPs. Cohen’s U3_1 displayed a large effect size for the AUC [1 (1 1)] and the amplitude [1 (0.5 1)]. **(C)** Representative traces. **(D)** Proof of concept: No eEPSPs after adding the AMPA-receptor antagonist NBQX. **(E)** s-ketamine led to no significant alteration of the relative time to peak (p = 0.156), nor to the relative decay time (*p* = 0.156) **(F)**.

We wanted to further characterize the effect of s-ketamine on NMDA-independent glutamatergic excitation. Therefore, we analyzed the AMPA-receptor-mediated intracortical synaptic transmission. The AUC of AMPA-receptor-mediated intracortical eEPSPs expanded significantly from 294 (119 748) pVms to 472 (134 1,159) pVms (*p* = 0.031). Cohen’s U3_1 indicated a large effect size [Cohen’s U3_1 = 1 (1 1)]. The amplitude enhanced from 5.2 (3.6 17.4) mV to 8.0 (3.9 25.8) mV (*p* = 0.063) with Cohen’s U3_1 equals 1 (0.5 1) (*n* = 6). Neither the time to peak nor the decay time (relative to control) was significantly altered [relative time to peak: control: 1 (1 1) vs. s-ketamine: 1.16 (0.90 1.56); *p* = 0.156; Cohen’s U3_1 = 0.3 (0 0.7); relative decay time: control: 1(1 1) vs. s-ketamine: 1.27 (0.82 1.78); *p* = 0.156; Cohen’s U3_1 = 0.3 (0 0.7); *n* = 6]. To prove, that the recordings display AMPA-mediated eEPSPs, we added the AMPA-receptor antagonist NBQX (5 μM), which extinguished the eEPSPs (Figure 8).

#### AMPA-receptor-mediated thalamocortical synaptic transmission

**Figure 9:**
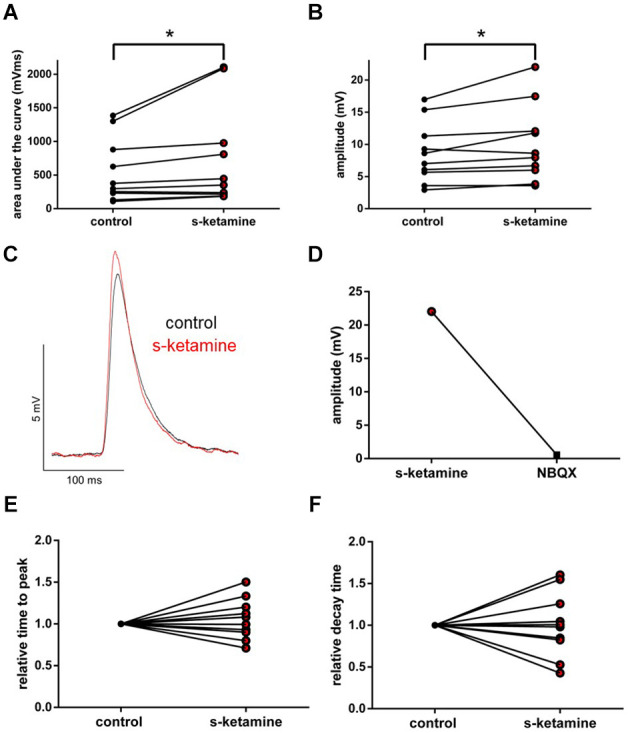
s-ketamine increased the AUC (*p* = 0.010) **(A)** and the amplitude (*p* = 0.014) **(B)** of thalamocortical AMPA-receptor mediated oEPSPs significantly (*). Cohen’s U3_1 of AUC was 0.8 (0.5 1) and 0.9 (0.7 1) of the amplitude. **(C)** Representative traces. **(D)** Proof of concept: No oEPSPs after adding the AMPA-receptor antagonist NBQX. **(E)** s-ketamine led to no significant alteration of the relative time to peak (*p* = 0.557), nor to the relative decay time (*p* = 0.999) **(F)**.

Under the influence of s-ketamine, a significant rise of the AUC (*p* = 0.010) from 337 (110 1,386) pVms to 399 (185 2,103) pVms and of the amplitude (*p* = 0.014) from 7.8 (2.9 17.0) mV to 8.3 (3.6 22.0) mV of AMPA-receptor-mediated thalamocortical oEPSPs was displayed. Cohen’s U3_1 indicated a large effect size of 0.9 (0.7 1) for the change of the amplitude and of 0.8 (0.5 1) for the AUC (*n* = 10). No significant alteration of the time to peak nor the decay time (relative to control) was observed [relative time to peak: control: 1 (1 1) vs. s-ketamine: 1.04 (0.71 1.50); *p* = 0.557; Cohen’s U3_1 = 0.5 (0.2 0.8); relative decay time: control 1(1 1) vs. s-ketamine: 0.99 (0.43 1.60); *p* = 0.999; Cohen’s U3_1 = 0.5 (0.2 0.8); *n* = 10]. Extinguishing oEPSPs by adding the AMPA-receptor antagonist NBQX (5 μM) to the perfusate proved the recorded oEPSPs to be AMPA-receptor-mediated (Figure 9).

## Discussion

In this study, we analyzed the effects of s-ketamine on the thalamocortical network in acutely prepared murine brain slices, using patch-clamp recordings in combination with optogenetic and electric stimulation. The thalamocortical and intracortical synaptic transmission were increased in the presence of s-ketamine. Depending on the subtype of ionotropic glutamate receptors, s-ketamine showed different actions. NMDA-receptor-mediated synaptic transmission was reduced, but interestingly, synaptic transmission driven by non-NMDA glutamate receptors was enhanced. A relevant attenuation of NMDA-receptor-dependent or independent spontaneous cortical inhibition was not observed. Moreover, intrinsic excitation properties of both pyramidal neurons in cortical layer IV and thalamocortical relay neurons in the VB remained essentially unaltered in our experiments.

EEG recordings display mainly cortical phenomena and result from changes in membrane potentials. While most anesthetics induce increased alpha oscillations, ketamine enhances gamma oscillations (Lee et al., 2013; Hemmings et al., 2019). Altered intrinsic neuronal excitability in cortical pyramidal neurons may be involved in this phenomenon. We, therefore, evaluated the effects of s-ketamine on the resting membrane potential, input resistance, membrane threshold for action potential generation, and tonic action potential frequency in pyramidal neurons of the cortical layer IV. Layer IV is the main target for input to the cortex and receives neuronal input both from cortical and subcortical projections. The input resistance and tonic action potential frequency remained largely unchanged. We found a statistically significant difference in the membrane threshold for action potential generation, but the effect size was ambiguous and a mean difference of 0.8 mV raises the question of actual physiological relevance. Likewise, the physiological relevance of altering the resting membrane potential for 0.6 mV is debatable, in particular as cortical hyperpolarization conflicts with raised activity. Hence, in a clinical context, we consider active and passive membrane biophysics of pyramidal neurons in cortical layer IV to be unaffected by s-ketamine. However, in accordance with the enhanced cortical electric activity, we did observe an increased intracortical synaptic transmission.

In order to experimentally replicate synaptic transmission, we used classic electric stimulation as well as optogenetic stimulation. Classic electric stimulation is suitable for smaller networks, like intracortical circuits. Though, in larger neuronal systems, like the thalamocortical network, high stimulation energy is required, which results in electric artifacts and electrothermal damage to the tissue. We, therefore, chose to incorporate an optogenetic stimulation for thalamocortical pathways and classic electric stimulation for intracortical pathways (Petreanu et al., 2007; Cruikshank et al., 2010). For the optogenetic experiments, an AAV was used, which is not able to replicate itself thus we reduced the expansion of infection and expression of ChR2 to neurons localized around the injection site, as depicted in Figure 1. Thereby we were able to reliably induce action potentials without adversely impairing intrinsic neuronal properties compared to electrical stimulation (Li et al., 2005). The ventrobasal thalamic complex as the injection site comprises only sporadic inhibitory interneurons and projects solely glutamatergic to the cortical lamina IV (Harris and Hendrickson, 1987; Feldmeyer, 2012). So optogenetic stimulation causes isolated excitatory thalamocortical synaptic transmission (Harris and Hendrickson, 1987). There is another advantage to this experimental approach: The VB is mainly a first order relay nucleus, but in a thalamocortical slice preparation, pathways of higher-order nuclei seem to be preserved (Lee and Sherman, 2008). Electric stimulation in the thalamus therefore could also stimulate unspecific higher-order nuclei. As we targeted the cortex with our laser for optogenetics, solely axons of ChR2 expressing excitatory thalamocortical relay neurons were excited.

Aside from monosynaptic input to the patched neuron, photo stimulation might lead to an excitation of indirect polysynaptic connections in the brain slice. In our experiments, we observed a rather fast onset of the potential following the photo stimulation. The latencies of our responses were around 5 ms. Furthermore, within the experiments, we did not observe a large variance of the latencies. For these two reasons, we are quite confident that we analyzed mono- rather than polysynaptic responses (Linders et al., 2022).

In cortical lamina IV, thalamic information is initially processed and then relayed to lamina II and III (Lübke and Feldmeyer, 2007). Beyond its gating function of relaying somatosensory information to the cortex, the thalamus also has a crucial role in corticocortical communication and further higher-order processing (Saalmann et al., 2012; Sherman, 2016; Hwang et al., 2017). Similar to other anesthetics, cortical connectivity appears to be impaired during ketamine-induced LOC (Blain-Moraes et al., 2014; Bonhomme et al., 2016; Vlisides et al., 2017). However, the cortical representation of somatosensory information is still preserved in the primary somatosensory cortex during ketamine anesthesia (Mashour, 2016; Schroeder et al., 2016). Consequently, it has been hypothesized, that ketamine causes LOC *via* impairment of higher cortical processing (Hudetz, 2012; Hudetz and Mashour, 2016; Mashour and Hudetz, 2018). Our results provide an *in vitro* correlation on a neuronal level to this approach. The thalamocortical synaptic transmission from the VB to the cortical lamina IV is preserved and even enhanced. The impaired cortical processing correlates with our findings of enhanced intracortical synaptic transmission.

Cortical activity patterns, as represented in EEG recordings, are severely influenced by thalamic activity. Hyperpolarization-activated cyclic nucleotide-gated cation channels (HCN), critically involved in thalamocortical oscillations, are inhibited by ketamine. Schnoebel et al. (2005) demonstrated, that ketamine blocks sodium and voltage dependent potassium channels in high concentrations. The observed effects could therefore also stem from altered neuronal excitability in thalamic relay neurons, but active and passive membrane biophysics of thalamocortical relay neurons in the thalamic ventrobasal complex remained unimpaired by s-ketamine.

Ketamine is a non-competitive NMDA-receptor antagonist. At resting membrane potential, the NMDA-receptor is physiologically blocked by Mg^2+^. This blockade is voltage-dependent, being dissolved when depolarized (Mayer et al., 1984; Nowak et al., 1984). We recorded NMDA-receptor-mediated events in voltage-clamp-controlled current-clamp mode. By setting the membrane potential to −40 mV, we dislodged the Mg^2+^-block and enabled recordings of NMDA-receptor-mediated EPSPs (Sutor et al., 2003). In our recordings, we were able to demonstrate, that s-ketamine acts as a NMDA-receptor antagonist by attenuating evoked NMDA-receptor-mediated intracortical synaptic transmission. This is in accordance with results from other groups (Anis et al., 1983; Coan and Collingridge, 1987; MacDonald et al., 1987; Davies et al., 1988; Orser et al., 1997).

It may not seem very intuitive that an anesthetic agent which acts as an antagonist of excitatory NMDA-receptors could generate excitation. NMDA-receptors on inhibitory interneurons are more active than the ones on principal neurons and are hence considered to be more sensitive to inhibition. Supposedly, the inhibition of inhibitory interneurons (i.e., disinhibition) subsequently causes excitation (Homayoun and Moghaddam, 2007; Seamans, 2008). We recorded GABA_A_-receptor-mediated inhibitory postsynaptic currents in pyramidal neurons of cortical lamina IV. Due to the application of specific receptor antagonists (and the consequent pharmacological isolation), the excitatory input to inhibitory interneurons was solely induced *via* NMDA-receptors. Surprisingly, the ascertaining of spontaneous NMDA-receptor-dependent GABA_A_-receptor-mediated inhibitory postsynaptic currents bore no evidence to confirm the aforementioned mode of disinhibition. In our recordings, there was no indication of reduced inhibition of pyramidal neurons, as one might expect.

Additionally, s-ketamine did not reduce spontaneous NMDA-receptor independent GABAergic inhibitory postsynaptic currents. There was a marginal but significant difference with regard to the frequency of IPSCs with an effect size indicating a medium to strong effect, which could lead to the hypothesis of a presynaptic effect of enhanced inhibition by cortical inhibitory interneurons. Yet it is questionable, whether these findings constitute a relevant aspect of s-ketamine’s effects. In any case, our experiments showed no evidence of an increased excitation through a reduction of inhibition. There is a potential GABA_A_ agonism assigned to ketamine but is only apparent in concentrations beyond clinical relevance (Flood and Krasowski, 2000; Gropper et al., 2020). Consistent with our findings, Wang et al. (2017) found no effect of ketamine in a similar concentration range on spontaneous postsynaptic GABA_A_ currents in murine hippocampal neurons. While we also recorded no effect on IPSC amplitude, there were differences with regard to IPSCS frequency. Similarly, Llamosas et al. (2019) reported no significant alteration of GABA_A_-receptor-mediated sIPSCs in the dorsal raphe nucleus under the influence of 50 μM ketamine. Ultimately, these recordings were performed in a quite heterogenous group of neurons and observed differences might be a reflection of this fact.

AMPA-receptors display a lower affinity towards glutamate but possess faster kinetics than NMDA-receptors. In physiological EPSPs, the fast, initial part is driven *via* AMPA-receptors (Meldrum, 2000). Here, we could demonstrate that s-ketamine increases AMPA-receptor-mediated intracortical synaptic transmission, while the NMDA-receptor-mediated intracortical synaptic transmission is reduced. Analogously, the AMPA-receptor-mediated thalamocortical synaptic transmission was also increased. As the thalamocortical pathway is exclusively glutamatergic, we conclude that the increased thalamocortical and intracortical excitation is based on increased AMPA-receptor-mediated excitation.

In contrast to our results, Yuan et al. (2016) showed a dose-dependent decrease in the amplitude of miniature and spontaneous postsynaptic glutamatergic currents in somatosensory cortical neurons of rats. As a limitation, in the aforementioned study fundamentally higher ketamine concentrations were used, likely in a range beyond clinical relevance (up to 1,000 μM). Ribeiro et al. (2014) reported no alteration of basal synaptic transmission in the hippocampus across a wide range of ketamine concentrations. According to Llamosas et al. (2019), 50 μM ketamine caused a raise in the frequency of AMPA-receptor-mediated sEPSCs in the dorsal raphe nucleus, but had no effect on the amplitude. Evoked AMPA-receptor-mediated EPSCs remained unaltered. In the ventral posteromedial nucleus, Fu et al. (2017) demonstrated that ketamine leads to a reduction of the amplitude of sEPSCs, but has no effect on the frequency. Further, miniature EPSCs remained unaltered. Directly comparing these results might pose a challenge, as evoked, spontaneous as well as miniature events are each shaped differently by pre- and postsynaptic mechanisms and hinge on specific experimental considerations (Sutton and Schuman, 2009; Chung et al., 2010).

In accordance with our findings, Nosyreva et al. (2013), who recorded evoked potentials in a more comparable concentration range as well as in a comparable exposure time to ketamine, demonstrated an increase of evoked AMPA-receptor-mediated field EPSPs after 30 min of 20 μM ketamine in the hippocampus of acute brain slices from rats. Further recordings of Autry et al. (2011) support these findings of increased evoked synaptic responses in field potentials after 30 min of 20 μM ketamine in the hippocampus.

In the case of neuropharmacological studies using acute brain slices, the application of concentrations with clinical relevance is of particular interest, as effects demonstrated in the presence of high concentrations might be unspecific in nature.

According to Idvall et al. (1979), the mean ketamine plasma concentration in humans during steady-state anesthesia is around 9.3 μM. According to Weber et al. (2004), the application of 2 mg s-ketamine/kg bodyweight in children leads to about 7.8 (±3.7) μM within 3 min. Bonhomme et al. (2016) described a plasma concentration of approximately 8.4 μM ketamine during the loss of consciousness (calculated with molar mass of 274.19). S-ketamine is the more effective enantiomer and is about two times as potent as the racemate ketamine (White et al., 1985; Ulrich Zeilhofer et al., 1992; Peltoniemi et al., 2016). Ketamine penetrates easily through the blood-brain barrier and accumulates fast in brain tissue (Cohen et al., 1973; Maxwell et al., 2006). Therefore, the concentration of 5 μM s-ketamine represents a clinically relevant concentration in our opinion.

Naturally, the results of this study should be interpreted in the light of its limitations. Firstly, acutely prepared brain slices offer—depending on the cutting specifications—relatively unscathed neuronal networks, that allow easy access to examine anesthetic effects on neuronal circuitry (Voss et al., 2019). On the downside, despite our efforts, a total absence of potential interference with our recordings cannot be guaranteed.

The use of exclusively female C57Bl/6N mice is a limitation of this study. Mice from postpartal days 28–35 have not reached full sexual maturity but cope with puberty. The mice used in experiments with optogenetic stimulation are up to 2 weeks older (postpartal days 35–49), so that there is a chance that some of these mice have reached estrus by then. The hormone profiles of the animals used were not determined. We cannot positively exclude potential hormonal, sex-specific interferences with our recordings (Whary et al., 2015). On a behavioral level, ketamine causes different effects in mice depending on the sex (Thelen et al., 2016). However, to our knowledge, sex-specific modulation on a neuronal level has not been reported with regard to s-ketamine, but also cannot be completely excluded.

A possible space clamp problem could also constitute another limitation. Recordings at the soma are restricted in clamping peripheral dendrites. Dendritic events and their possible impact stay underrepresented by using a potassium-based intracellular solution.

While optogenetic stimulation itself as well as the injection of the applied AAV are established methods for electrophysiologic investigations and have been used for ketamine recordings previously, we cannot ensure that the ChR2 is completely inert to s-ketamine. This constitutes a possible limitation.

In conclusion, we found no evidence that s-ketamine is able to induce NMDA-receptor-dependent spontaneous cortical disinhibition or to reduce NMDA-receptor independent spontaneous cortical inhibition. We confirmed s-ketamine’s NMDA-receptor antagonism in evoked synaptic transmission. While the intrinsic excitability of cortical pyramidal neurons in lamina IV and thalamocortical relay neurons in the VB was not substantially affected, we could demonstrate that s-ketamine enhances AMPA-receptor-mediated thalamocortical synaptic transmission as well as AMPA-receptor-mediated intracortical synaptic transmission. Therefore, the increased thalamocortical and intracortical synaptic transmission appears to be driven predominately *via* AMPA-receptor pathways.

## Data Availability Statement

The raw data supporting the conclusions of this article will be made available by the authors, without undue reservation.

## Ethics Statement

The animal study was reviewed and approved by Ethical Committee on Animal Care and Use of the Government of Bavaria (Munich, Germany).

## Author Contributions

MB conducted experiments, performed data analysis, and wrote the manuscript. SS provided critical feedback on the manuscript. MK designed analysis and provided critical feedback on the manuscript. CK assisted in experiments and data analysis. MH performed experiments and data analysis. GS provided critical feedback on the manuscript. RH and SK designed study and analysis, surveyed interpretation and discussion, contributed to writing the manuscript, and critical feedback. All authors contributed to the article and approved the submitted version.
